# Using random forests to model 90-day hometime in people with stroke

**DOI:** 10.1186/s12874-021-01289-8

**Published:** 2021-05-10

**Authors:** Jessalyn K. Holodinsky, Amy Y. X. Yu, Moira K. Kapral, Peter C. Austin

**Affiliations:** 1 Department of Clinical Neurosciences, Cumming School of Medicine, University of Calgary, 3330 Hospital Drive NW, Calgary, AB T2N4N1 Canada; 2 ICES, Toronto, ON Canada; 3 Department of Medicine (Neurology), University of Toronto, Sunnybrook Health Sciences Centre, Toronto, ON Canada; 4 Department of Medicine (General Internal Medicine), University of Toronto and University Health Network, Toronto, ON Canada; 5 Institute of Health Policy, Management, and Evaluation, University of Toronto, Toronto, ON Canada; 6 Schulich Heart Research Program, Sunnybrook Research Institute, Toronto, ON Canada

**Keywords:** Stroke, Hometime, Random forests

## Abstract

**Background:**

Ninety-day hometime, the number of days a patient is living in the community in the first 90 after stroke, exhibits a non-normal bucket-shaped distribution, with lower and upper constraints making its analysis difficult. In this proof-of-concept study we evaluated the performance of random forests regression in the analysis of hometime.

**Methods:**

Using administrative data we identified stroke hospitalizations between 2010 and 2017 in Ontario, Canada. We used random forests regression to predict 90-day hometime using 15 covariates. Model accuracy was determined using the *r-squared* statistic. Variable importance in prediction and the marginal effects of each covariate were explored.

**Results:**

We identified 75,745 eligible patients. Median 90-day hometime was 59 days (Q1: 2, Q3: 83). Random forests predicted hometime with reasonable accuracy (adjusted r-squared 0.3462); no implausible values were predicted but extreme values were predicted with low accuracy. Frailty, stroke severity, and age exhibited inverse non-linear relationships with hometime and patients arriving by ambulance had less hometime than those who did not.

**Conclusions:**

Random forests may be a useful method for analyzing 90-day hometime and capturing the complex non-linear relationships which exist between predictors and hometime. Future work should compare random forests to other models and focus on improving the accuracy of predictions of extreme values of hometime.

**Supplementary Information:**

The online version contains supplementary material available at 10.1186/s12874-021-01289-8.

## Background

Stroke is a leading cause of morbidity and mortality worldwide. Assessing patient outcome after stroke is important for clinical research and quality improvement initiatives. Post-stroke recovery is commonly determined using scales or questionnaires delivered via structured interview. There are several common scales available to measure outcomes after stroke, the most common being the modified Rankin Scale. However, scales such as this are subject to issue with rater bias, inter-observer variability, social desirability bias in self-reporting, and attrition bias [1]. Additionally, these scales require prospective evaluation by trained experts and as such are not commonly collected outside the clinical trial environment, meaning they cannot be used for population based studies nor for retrospective observational studies. Ninety-day hometime, defined as the total number of days a patient is living in the community (and not in a healthcare institution) in the first 90 days after stroke [2], is a new stroke outcome metric shown to be correlated with disability after stroke [3–7]. Hometime is objective and does not suffer from inter/intra-rater reliability issues or any issues related to self-reporting. Hometime can be obtained from administrative data, enabling population-based analyses [5]. Hometime is graded, with longer home-time being associated with higher post-stroke disability [2–6], unlike other outcomes available in administrative data such as mortality. Finally, home-time is meaningful to patients because they value reintegration into the community after stroke as well as policy-makers because this metric is intuitively associated with healthcare costs [8–10].

In prior studies, a substantial range of statistical methods have been used to analyze hometime including negative binomial regression, ordinal logistic regression, median regression, linear regression, Spearman rank correlation, t-test and chi-square analyses, propensity score matching, and categorizing hometime into quartiles [3–7, 11–13]. While this diversity may be due to the individual study objectives, it may also reflect unique statistical properties of hometime, which make its analysis problematic. Indeed, typical parametric statistical methods may have limited utility for analyzing hometime because it follows a highly non-normal bucket shaped distribution with spikes at or near its lower and upper limits (by design, 90-day hometime is constrained to lie between 0 and 90) [5, 6] Further, the lower and upper limits themselves cause additional difficulty with applying traditional regression methods to predict hometime, as they may result in non-plausible estimated values, such as estimating a negative hometime or extrapolating beyond 90 days.

Given these challenges, random forests regression, a popular method from the machine learning literature, may be a more suitable method for the analysis of hometime. We aimed to study the use of random forests regression for modelling 90-day hometime in a population-based cohort of stroke patients, and to determine the relative importance of several covariates in the prediction of hometime using random forests regression. We have focused this paper solely on random forests regression as a proof of concept illustrating the utility of random forests for hometime. We do not compare the use of random forests to other regression methods in this paper. We have structured this article in the following way: first, we describe random forests regression and its advantages for analyzing hometime. Second, we apply random forests regression to predict 90-day hometime in a population-based cohort of stroke patients and discuss the model’s performance. Third, we highlight the relative importance of several clinically relevant covariates in the prediction of hometime using this method.

## Random forests

### Overview of random forests

Classification and regression trees (CART) are a simple tool for prediction and classification. Unlike linear regression, CART is not based on a parametric regression model, but rather data are split along the predictor axes into groups (nodes). A node is split on the variable that results in the two resultant sub-nodes being as homogeneous as possible [14]. This process is then repeated recursively with each of the two resultant sub-nodes. Predictors can be categorical or continuous (using a data-driven cut-off value for the split); outcomes can also be categorical or continuous. Random forests is a regression method based on the aggregation of a large number of these trees which has been shown to produce more accurate results than just a single tree [15]. A random forest is a variation of bootstrap aggregating (bagging) where several hundred trees are created from the same dataset and their results averaged. The training data for each tree is created from a bootstrapped sample of the full dataset, meaning that approximately one third of the observations will not be used in the training dataset. Each time a split is considered, a random sample of the predictors (among the full set of predictors) are chosen as candidates for the split. This allows multicollinearity to be handled as not all predictors are considered at each split [15]. Trees are grown to maximum size without pruning. The predictions for each observation obtained from each tree are averaged.

There are several advantages to this methodology. First, single trees can be prone to overfitting and are very sensitive to small changes in the training data [16]. Second, through bagging, there are data points which do not end up in the bootstrapped sample for any given tree (out-of-bag observations); this allows for a statistically efficient process where the random forest can be fit in one sequence with cross validation being performed along the way [16]. Finally, this methodology allows the model to capture complex interaction structures within the data in with relatively low bias [15].

One disadvantage is that this method does not produce regression coefficients which allow for the direct interpretation of each variable’s impact on the outcome of interest [17]. However, by measuring the effect of variable permutation on the model’s accuracy (measured using out-of-bag error estimation) and node homogeneity (measured using the Gini index), random forests allow for a variable importance measure to be determined for each predictor. As a result, one can tell, relative to the other predictors, each variable’s importance in prediction of the outcome. We have provided more information on the procedures for assessing variable importance in the supplemental materials. There are also other model-agnostic interpretation methods, such as partial dependence, which allow for examining the marginal effects of each variable (one or two at a time) on the model’s predictions.

### Advantages of random forests for the analysis of Hometime

One of the biggest advantages of random forests is that they do not make any distributional assumptions about underlying data structures, meaning they can be used on data which exhibiting highly unusual distributions, such as those in hometime. One property of regression trees, sometimes discussed as a limitation, is that they cannot perform extrapolation. The estimates produced are constrained to averages of the observed data; meaning predictions which are less than the minimum or greater than the maximum outcome value which appears in the dataset on which the model was trained cannot be obtained [17]. In the setting of 90-day hometime, this is an advantage, as the random forest cannot produce non-plausible estimated values of hometime (those < 0 or > 90 days).

## Methods

### Cohort identification

Using the Canadian Institute for Health Information (CIHI) Discharge Abstract Database (DAD) we identified all patients with a main diagnosis of stroke (ischemic or intracerebral hemorrhage) admitted to an acute care hospital in Ontario between April 1, 2010 and December 31, 2017. Nonresidents of Ontario, those < 18 or > 105 years of age, strokes occurring in-hospital, patients discharged from the emergency department without in-patient hospitalization, patients with history of prior stroke, and patients in long-term care at baseline were excluded.

### Covariates

Covariates of interest included age, sex, arrival by ambulance, stroke type, treatment with thrombolysis, stroke unit care, frailty (measured using the Hospital Frailty Risk Score, a continuous score from 0 to 99 derived from administrative data where scores < 5 indicate low risk of frailty, scores 5–15 indicate moderate risk of frailty and scores > 15 indicate high risk of frailty) [18], stroke severity (measured using the Passive Surveillance Stroke seVerity Indicator (PaSSV) where scores < 4 indicate severe stroke, scores 4–8 indicate moderate stroke severity, and scores > 8 indicates mild stroke severity) [19], rural vs. urban home location, quintile of median neighbourhood income, and the following comorbidities: atrial fibrillation, diabetes, hypertension, myocardial infarction. A 5-year lookback window was used for all comorbidities. Covariates were identified using linked data from the DAD, the Ontario Health Insurance Plan Database, the Ontario Diabetes Dataset [20, 21], the Ontario Hypertension Dataset [22, 23], the Ontario Myocardial Infarction Dataset [24], and the Canadian Census (case definitions are given in Table A.1). Patients with missing data were excluded from the analyses.

### Ninety-day Hometime calculation

We calculated 90-day hometime using linked data from the following sources: DAD (inpatient hospitalization), National Ambulatory Care Reporting System (emergency department), the National Rehabilitation Reporting System (rehabilitation), the Continuing Care Reporting System (complex continuing care or long-term care), and the Ontario Registered Persons Database (mortality data). Data linkage occurred through unique encoded identifiers at ICES; these datasets have been validated extensively for research purposes [25].

For patients who survived to day 90, 90-day hometime was calculated as 90 minus the sum of length(s) of stay in ED, acute care, rehabilitation, and long-term care. For example, a patient whose sum of lengths of stay in healthcare institutions = 20 days would have a hometime of 70 days. Patients who died prior to day 90 could still accumulate hometime days for each day spent alive and out of healthcare institutions prior to death. For example, a patient with whose sum of lengths of stay in healthcare institutions = 20 days and died on day 70 would have a hometime of 50 days. Patients who died during the index admission have, by definition, hometime of 0 days. Hometime accumulation does not have to be continuous. For example, a patient with an acute care admission who was discharged to home and then re-admitted within 90 days of index event would have both admission lengths of stay subtracted for the 90-day hometime calculation.

### Statistical methods

We used random forests regression to model 90-day hometime. A random forest consisting of 500 trees was grown, using p/3 candidate predictors at each split (where p = total number of predictors) in accordance with recommendations made by Breiman [15]. All trees were grown using a minimum node size of 5 and no restrictions on tree depth or number of terminal nodes were imposed. Model fit was assessed using adjusted R-squared.

Using both out-of-bag error estimation and node homogeneity, the relative importance of each co-variate in predicting hometime was determined. The marginal effects that each co-variate had on the predicted outcome were illustrated using partial dependence plots. These plots show how predicted values *partially depend* on the values of one or more co-variates. These graphs plot the change in average predicted outcome value as a co-variate is varied over its marginal distribution [26]. These plots are post-hoc methods of model interpretation, they do not reveal the inner workings of the model, but rather reveal how the model behaves as a result of changing inputs. One-way partial dependence plots were generated for each co-variate. Two-way partial dependence plots were generated to depict the interaction between pairs of variables that displayed high importance for hometime. All analyses were performed using R (v3.3.0).

### Ethics and data availability statement

This study was approved by the Sunnybrook Health Sciences Centre Research Ethics Board. The use of data in this project was authorized under section 45 of Ontario’s Personal Health Information Protection Act. The data sets used for this study were held securely in a linked, de-identified form and analyzed at ICES. While data sharing agreements prohibit ICES from making the data set publicly available, access may be granted to those who meet pre-specified criteria for confidential access, available at www.ices.on.ca/DAS.

## Results

### Patient characteristics

**Fig. 1:**
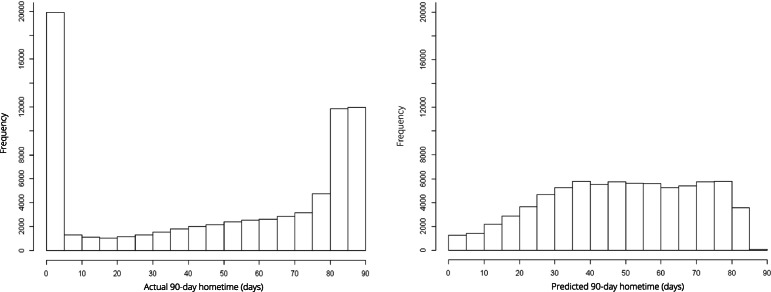
Left: Histogram of 90-day hometime across the cohort of 75,475 stroke patients. Right: Histogram of predicted 90-day hometime across 75,475 patients using a random forests model with 15 clinically relevant covariates

From 109,842 acute admissions for stroke, we identified a cohort of 75,475 patients with complete data who met all inclusion criteria. The cohort selection flow chart is presented in Figure A1. We removed 202 observations with small cell counts upon cross tabulation of baseline characteristics to avoid potential re-identification of individuals as per ICES policy; aggregate demographics of these patients are given in Table A.2. Baseline characteristics of the final cohort are given in Table 1. At Day 90, 68.54% of patients were home and 17.49% of patients had died. The distribution of 90-day hometime across the entire cohort of patients is displayed in Fig. 1. The median 90-day hometime across the cohort was 59 days (Q1: 2, Q3: 83). The pairwise correlation between all covariates is given in Table A.3. Some of the predictors exhibited moderate correlation with the highest magnitude being between PaSSV score and admission via ambulance (*ρ* = − 0.45); however, as random forests regression is robust to multicollinearity all variables were included as candidates in the model.

**Table 1:** Baseline characteristics of patients hospitalized with acute stroke between April 1, 2010 and December 31, 2017 and included in the study cohort

Characteristic	Complete Case Analysis Cohort (***n*** = 75,475)
Female (%)	47.44
Median Age (Q1, Q3) - years	75 (64, 84)
Arrived by Ambulance (%)	71.19
Stroke Type (%)	
Intra-cerebral hemorrhage	12.87
Ischemic Stroke	87.12
Diabetes (%)	36.61
Atrial Fibrillation (%)	14.18
Hypertension (%)	82.76
Myocardial Infarction (%)	9.19
Neighbourhood Income Quintile (%)	
Quintile 1 (lowest)	23.60
Quintile 2	21.99
Quintile 3	19.70
Quintile 4	17.75
Quintile 5 (highest)	16.96
Home Location (%)	
Rural	12.40
Urban	87.60
Median Frailty Score^a^ (Q1, Q3)	4.2 (0.8, 9.1)
Median PaSSV Score^b^ (Q1, Q3)	7.7 (6.5, 8.7)
Received Thrombolysis (%)	13.36
Received Stroke Unit Care (%)	56.01

Q1: first quartile; Q3: third quartile; PaSSV: Passive Surveillance Stroke seVerity indicator

^a^A continuous score ranging from 0 to 99 where scores < 5 indicate low risk of frailty, scores from 5 to 15 indicate intermediate risk of frailty, and scores > 15 indicate high risk of frailty [18]

^b^A continuous score where < 4 indicates severe stroke, 4–8 indicates moderate stroke severity, and > 8 indicates mild stroke severity [19]

### Using random forests regression to predict 90-day Hometime

The random forests model predicted 90-day hometime with reasonable accuracy (adjusted r-squared = 0.3462). The distribution of predicted hometime across the cohort is displayed in Fig. 1. Extreme values of hometime, both low and high, were predicted with the least accuracy. Low hometime values were systematically over-estimated and high hometime values were systematically under-estimated (Figure A.2). All predicted values for hometime were plausible (minimum: 0 days; maximum: 87.39 days).

### Interpretation of random forests model

**Fig. 2:**
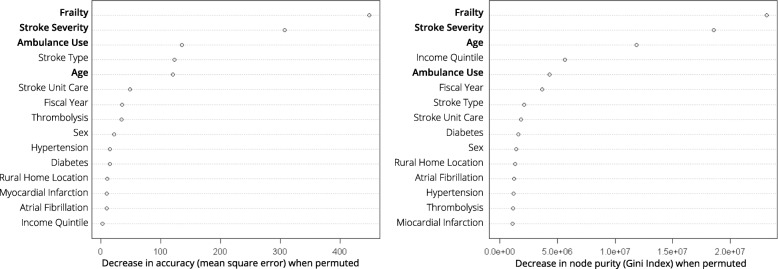
Variable importance plots for random forest model using 15 covariates to predict hometime in a cohort of 75,475 stroke patients. Four among the top five most important variables (bold face) were the same across both methods

Whether determining variable importance using model accuracy (out-of-bag error estimation) or node purity (Gini index), four of the top five ranked variables were the same: frailty, stroke severity, age, and ambulance use (Fig. 2). The two top predictors, frailty and stroke severity, were the same for both methods of ranking variable importance and on a relative scale these variables were far more important than the other 13 covariates in predicting hometime.

### Influence of individual covariates on Hometime predictions

Using both one and two-way partial dependence plots, we examined the relationships between the four co-variates ranked of high importance in predicting hometime. These partial dependence plots are interpreted as the relationship between the predictor variable(s) and 90-day hometime after averaging out the effects of all other predictors. The partial dependence estimates of the other 11 variables of relatively lower importance are summarized in Table 2.

**Table 2:** Marginal estimate of hometime for variables with lower relative importance on predicted hometime

Variable	Predicted 90-day hometime (days)
Sex	
Female	48.8
Male	48.7
Stroke Type	
Intra-cerebral Hemorrhage	40.0
Ischemic Stroke	50.0
Diabetes	
Yes	47.7
No	49.3
Atrial Fibrillation	
Yes	48.5
No	48.8
Hypertension	
Yes	48.8
No	48.3
Myocardial Infarction	
Yes	49.3
No	48.7
Neighbourhood Income Quintile	
Quintile 1 (lowest)	47.9
Quintile 2	48.6
Quintile 3	49.2
Quintile 4	49.0
Quintile 5 (highest)	49.2
Home Location	
Rural	47.8
Urban	49.0
Received Thrombolysis	
Yes	48.5
No	48.5
Received Stroke Unit Care	
Yes	49.7
No	47.5
Fiscal Year Group	
2010–2011	45.4
2012–2013	46.8
2014–2015	49.6
2016–2017	51.1

**Fig. 3:**
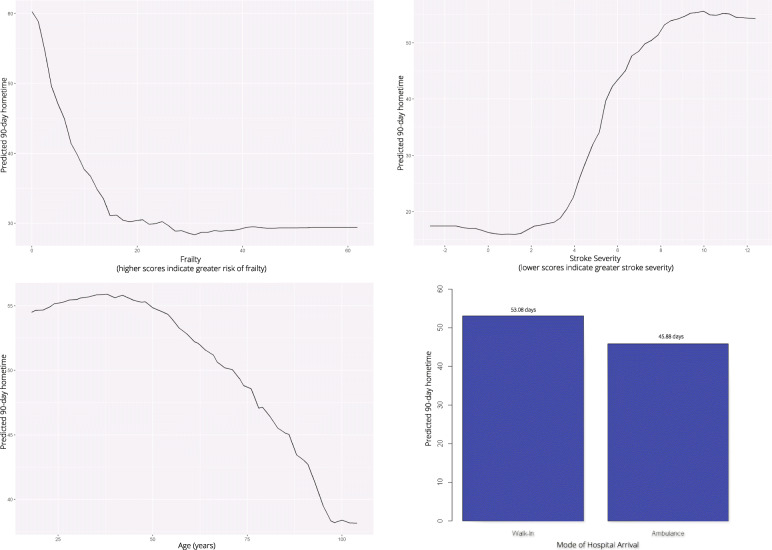
Partial dependence plots illustrating the effect of frailty (top left), stroke severity (measured using the PaSSV score) (top right), age (bottom left), and ambulance use (bottom right) on predicted 90-day hometime averaging out the effects of all other predictors. Frailty score is a continuous score from 0 to 99 derived from administrative data where scores < 5 indicate low risk of frailty, scores 5–15 indicate moderate risk of frailty and scores > 15 indicate high risk of frailty [18]. Passive Surveillance Stroke seVerity Indicator (PaSSV) score is a continuous score calculated from administrative data where scores < 4 indicate severe stroke, scores 4–8 indicate moderate stroke severity, and scores > 8 indicates mild stroke severity [19]

**Fig. 4:**
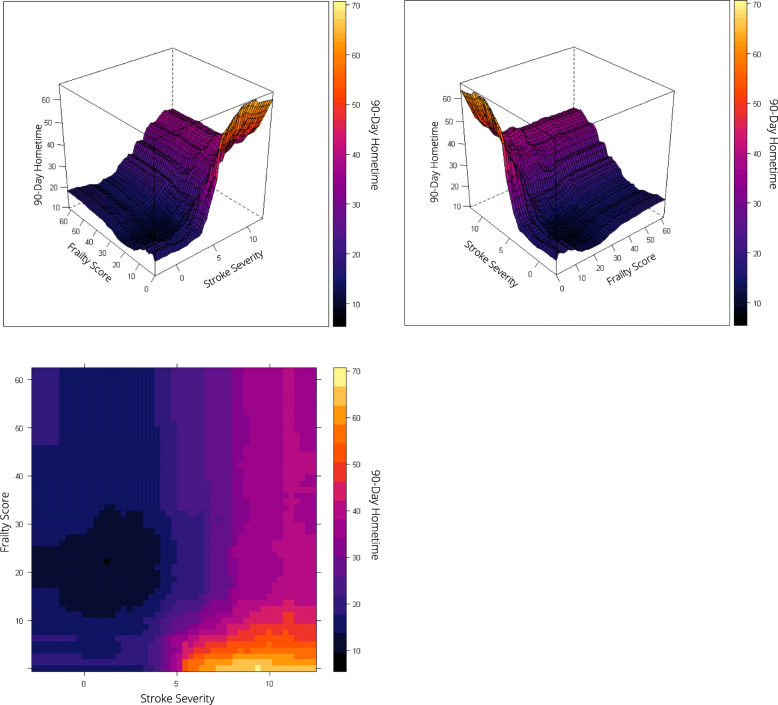
Two-way partial dependence plots depicting the relationship between frailty, stroke severity, and predicted 90-day hometime. The top two plots depict this relationship in a three-dimensional plot viewed from two different perspectives with predicted hometime displayed on the z-axis. The bottom plot displays this relationship using a two-dimensional contour plot. All three plots use the same color scale to represent predicted 90-day hometime where darker colors indicate less hometime. Frailty scores range from 0 to 99 with higher scores indicating greater risk of frailty (scores < 5 indicate low risk of frailty, scores 5–15 indicate moderate risk of frailty and scores > 15 indicate high risk of frailty) [18]. Stroke severity is measured using the Passive Surveillance Stroke seVerity Indicator (PaSSV) where scores < 4 indicate severe stroke, scores 4–8 indicate moderate stroke severity, and scores > 8 indicates mild stroke severity [19]

Frailty and stroke severity were the top predictors of hometime, and the associations were non-linear. For patients with low or moderate risk of frailty (scores ≤15), as frailty increased predicted hometime decreased; however, for patients at high risk of frailty (scores > 15), there was little change in predicted hometime as frailty score increased (Fig. 3). Predicted hometime increased as stroke severity decreased, but there was less variability in predicted hometime for those with high or low stroke severity compared to those with moderate stroke severity (Fig. 3). There was an interaction between frailty and stroke severity; the rapid decrease in hometime with increasing frailty was only seen when stroke severity was low (Fig. 4). For higher stroke severity, estimated hometime remained relatively constant regardless of frailty.

**Fig. 5:**
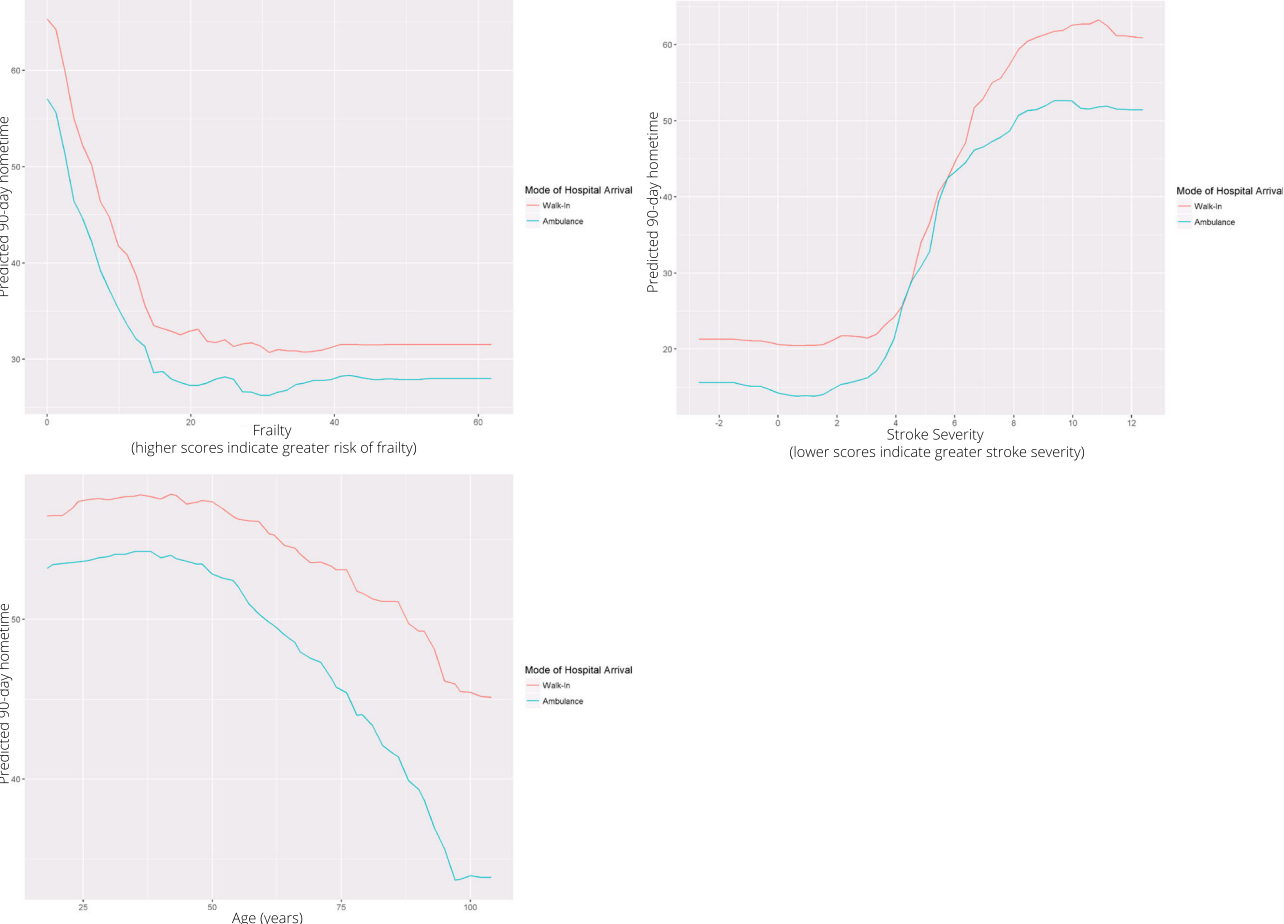
Top left – Two-way partial dependence plot depicting the relationship between frailty and 90-day hometime stratified by ambulance use. Top right – Two-way partial dependence plot depicting the relationship between stroke severity and 90-day hometime stratified by ambulance use. Bottom – Two-way partial dependence plot depicting the relationship between age and 90-day hometime stratified by ambulance use. Frailty scores range from 0 to 99 with higher scores indicating greater risk of frailty (scores < 5 indicate low risk of frailty, scores 5–15 indicate moderate risk of frailty and scores > 15 indicate high risk of frailty) [18]. Stroke severity is measured using the Passive Surveillance Stroke seVerity Indicator (PaSSV) where scores < 4 indicate severe stroke, scores 4–8 indicate moderate stroke severity, and scores > 8 indicates mild stroke severity [19]

Patients who arrived by ambulance had lower predicted hometime than those who did not (45.9 vs. 53.1 days) (Fig. 3). Arrival by ambulance did not change the nature of the association between hometime and frailty or stroke severity, but it created a downward shift as patients arriving by ambulance overall had less predicted hometime than those who did not (Fig. 5).

**Fig. 6:**
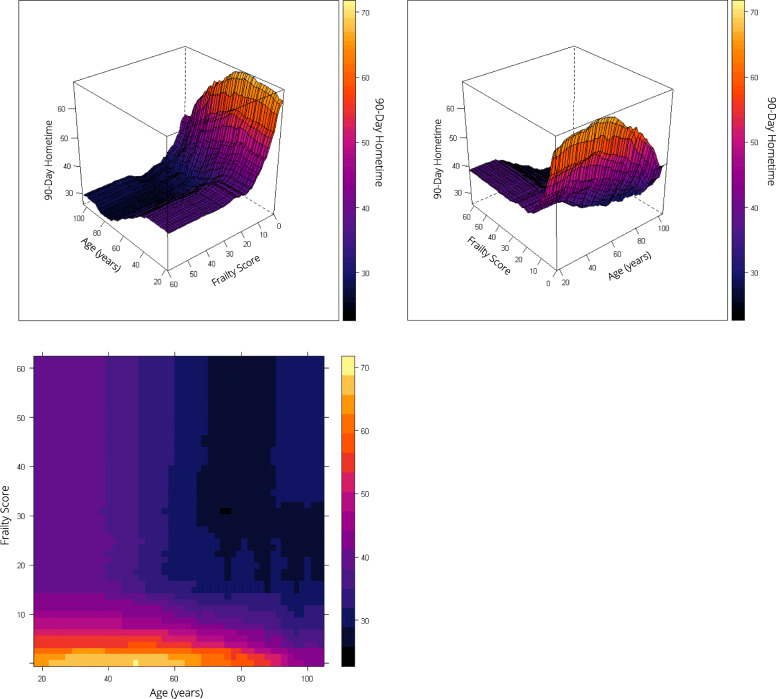
Two-way partial dependence plots depicting the relationship between frailty, age, and predicted 90-day hometime. The top two plots depict this relationship in a three-dimensional plot viewed from two different perspectives with predicted hometime displayed on the z-axis. The bottom plot displays this relationship using a two-dimensional contour plot. All three plots use the same color scale to represent predicted 90-day hometime where darker colors indicate less hometime. Frailty scores range from 0 to 99 with higher scores indicating greater risk of frailty (scores < 5 indicate low risk of frailty, scores 5–15 indicate moderate risk of frailty and scores > 15 indicate high risk of frailty) [18]

**Fig. 7:**
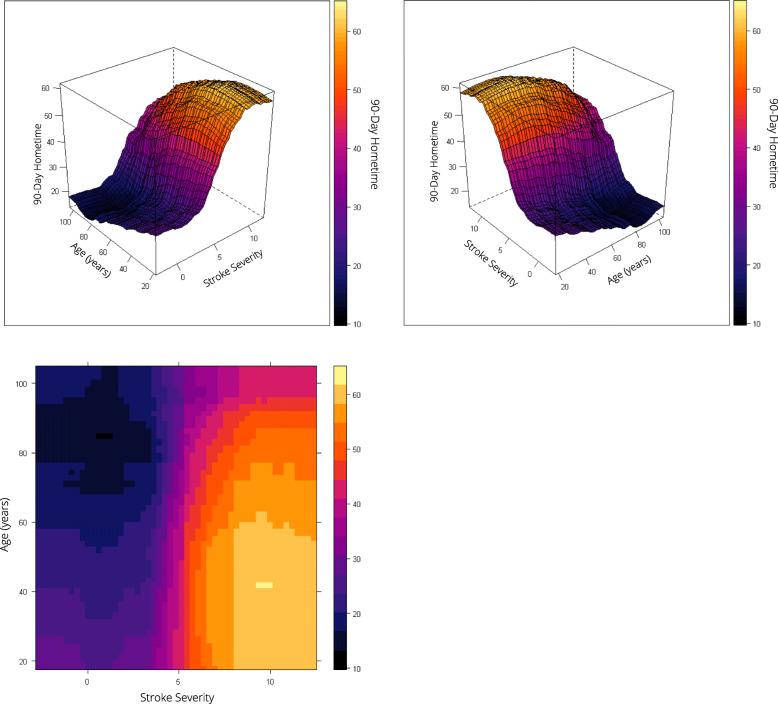
Two-way partial dependence plots depicting the relationship between stroke severity, age, and predicted 90-day hometime. The top two plots depict this relationship in a three-dimensional plot viewed from two different perspectives with predicted hometime displayed on the z-axis. The bottom plot displays this relationship using a two-dimensional contour plot. All three plots use the same color scale to represent predicted 90-day hometime where darker colors indicate less hometime. Stroke severity is measured using the Passive Surveillance Stroke seVerity Indicator (PaSSV) where scores < 4 indicate severe stroke, scores 4–8 indicate moderate stroke severity, and scores > 8 indicates mild stroke severity [19]

Age displayed a non-linear relationship with hometime, with predicted hometime decreasing with increasing age, especially beyond age 45 (Fig. 3). The rapid decrease and then plateau in hometime as frailty increased held true across all ages (Fig. 6). The S-shaped relationship between hometime and stroke severity also persisted across all ages (Fig. 7). Patients presenting via ambulance had less hometime than those who did not across all ages, but the difference in predicted hometime between the two groups increased with age (Fig. 4).

## Discussion

We found that a random forests regression model predicts hometime with reasonable accuracy without predicting implausible values. The random forests model allowed for the capturing and describing complex non-linear relationships between predictors and hometime, such as for frailty and stroke severity.

However, patients with extreme values of hometime were systematically under predicted, especially those with 0-hometime. This could be because there are two distinct groups of patients with hometime of 0 days: 1) those who did not survive the initial stroke admission and 2) those who survived with severe disability and remained institutionalized for the full duration of the 90 days. The characteristics of these two groups may be different and using a single model to predict these outcomes may not be ideal. Interestingly, the model also systematically under predicted hometime values for patients with high hometime. Unlike 0-hometime, high hometime only has one interpretation, that the patient was sufficiently well for early discharge to home. Another potential reason for the suboptimal prediction of the extreme values of hometime is that our set of potential covariates did not include variables which could be associated with both going home quickly and not returning home at all, such as marital status, living situation, lifestyle factors, social support, and indicators of quality of care, as these are not available in administrative data.

We found that the most important variables for predicting hometime were frailty, stroke severity, age, and ambulance use. Our findings are consistent with prior work showing that frailty [27], stroke severity, [12, 13] and age [6, 12, 13] are associated with disability after stroke, but the association between these variables and hometime specifically is not yet well understood.

Our findings of patient location (rural vs. urban) being relatively unimportant was consistent with previous literature [6, 12]. We found that patients with intracerebral hemorrhage had 10.0 fewer days of hometime than patients with ischemic stroke, also consistent with previous literature [6, 13]. Sex was not associated with hometime, which has been previously reported by some studies [5] but not others [6, 12]. We did not see a difference in hometime based on thrombolysis use in this study. Prior work has shown patients receiving thrombolysis have increased hometime [3]; however, this previous study focused on patients with acute ischemic stroke who were eligible for thrombolysis whereas our study included hemorrhagic stroke patients and ischemic stroke patients who may not have been eligible for thrombolysis. Individual vascular comorbidities (atrial fibrillation, diabetes, myocardial infarction, hypertension) were not associated with hometime, suggesting that multi-morbidity, as captured by the frailty score, is likely more important in predicting outcomes after stroke than any specific comorbidity. This is consistent with our understanding of the effects of multi-morbidity on stroke outcomes [28, 29].

There are limitations to using random forests. Random forests are complex, consisting of hundreds of regression trees. This means that 1) a large amount of computation power and time are needed to generate them, and 2) they don’t produce readily interpretable coefficients like those produced in linear regression or other parametric models. We have used variable importance and partial dependence plots to assist in model interpretability and assess the marginal effects of each covariate. There are other methods available to assess variable importance and marginal effects of covariates including SHAP plots, LIME plots, and global surrogates which were not explored in this paper [30]. Finally, it is important to be aware that random forests cannot perform extrapolation. While this is an advantage for a bounded outcome like hometime, as they will not generate implausible predictions, it can be a limitation if the range of outcome values in the test set is larger than that in the training set.

## Conclusion

Random forests regression may be a useful analytic method for predicting 90-day hometime, a bounded variable with a highly non-normal distribution. The random forests regression model was able to capture complex non-linear relationships as well as interactions between many important covariates and hometime. Predictive accuracy was lowest for extreme values of hometime which may warrant future study. Future work should also focus on the comparison of random forests to other models.

## Supplementary Information

**Additional file 1: Table A.1.** Administrative data comorbidity case definitions. **Table A.2.** Demographics of patients excluded due to privacy issues. **Table A.3.** Pairwise correlation of all covariates. **Figure A.1.** Cohort selection. **Figure 2.** Random forests model residuals compared to actual 90-day hometime values.

## Data Availability

The data sets used for this study were held securely in a linked, de-identified form and analyzed at ICES. While data sharing agreements prohibit ICES from making the data set publicly available, access may be granted to those who meet pre-specified criteria for confidential access, available at www.ices.on.ca/DAS.

## Abbreviations

CART: Classification and regression tree
Bagging/ed: Bootstrap aggregation/ed
CIHI: Canadian Institute for Health Information
DAD: Discharge Abstract Database
PaSSV: Passive Surveillance Stroke seVerity Indicator
ED: Emergency department
