# Supplementary material for: Using random forests to model 90-day hometime in people with stroke
Source: BMC Med Res Methodol. 2021 May 10;21:102. doi: 10.1186/s12874-021-01289-8 (PMC8112132; doi:10.1186/s12874-021-01289-8)
Supplement: Supplementary file 1 — Additional file 1: Table A.1. Administrative data comorbidity case definitions. Table A.2. Demographics of patients excluded due to privacy issues. Table A.3. Pairwise correlation of all covariates. Figure A.1. Cohort selection. Figure 2. Random forests model residuals compared to actual 90-day hometime values. [file 12874_2021_1289_MOESM1_ESM.docx]

**Additional File 1**

Using random forests to model 90-day hometime in people with stroke

Jessalyn K. Holodinsky, PhD, Amy Y.X. Yu, MD MSc, Moira K. Kapral, MD MSc, Peter C. Austin, PhD

**Table of Contents**

**Supplemental Methods**

**Table A.1.** Administrative data comorbidity case definitions

**Table A.2.** Demographics of patients excluded due to privacy issues

**Table A.3.** Pairwise correlation of all covariates

**Figure A.1.** Cohort selection

**Figure A.2.** Random forests model residuals compared to actual 90-day hometime values

**Supplemental Methods**

*Measuring Variable Importance*

In random forest models, variable importance (defined as the contribution the variable makes to model performance) can be determined using two different metrics. The first being accuracy as measured by out-of-bag error. As each individual tree is generated from a bootstrapped data set approximately one third of the data points will not be used in the training set for any given tree – these are known as the out-of-bag observations. As they are not used in the training set for tree generation they can be used as test observations to determine the trees accuracy. After generation of the random forest the out-of-bag observations can be run through their respective decision trees and the predicted outcome for these observations recorded. These predicted outcomes are compared to the actual outcome and the collective mean squared error in the predictions is calculated – this is known as the out-of-bag error rate.[1]

The out-of-bag observations and the mean square error rate from the random forests model can be used to determine variable importance through variable permutation. In this method, the values of a variable are randomly permuted one at a time (with all other variables fixed) in the out-of-bag datasets. This new out-of-bag data are passed down their respective trees and new predictions and mean square error calculated. This new out-of-bag error rate will almost certainly be higher than the out-of-bag error rate for the original data set. The data values are then reset and the process repeated with each variable. The variable which results in the greatest increase in out-of-bag error rate when permuted (relative to the other variables) is the most important in predicting the outcome. The variables which result in the least increase in out-of-bag error rate when permuted (relative to the other variables) are the least important in predicting the outcome.[1-3]

As an example, say patient age was one of the variables of interest, the patient’s age in each out-of-bag observation would be changed to new, plausible, but random values. This new out-of-bag data with random values for patient age would be run through the random forest and the predicted outcomes and mean square error recorded. If age was a very important predictor in the outcome (relative to the other variables in the dataset) we would expect that permuting its values would cause large changes in predicted outcome and as such a large increase in out-of-bag mean square error. However, if age is unimportant in predicting the outcome then we would expect little change in both the predicted outcome and out-of-bag mean square error. If this process is repeated for all variables in the dataset then, relative to one another, it is determined which is most important in predicting the outcome of interest.

The second way to measure variable importance is through the Gini index which is a measure of how each variable contributes to node homogeneity. When creating the individual trees in the random forest the goal is to create trees with nodes which are as homogeneous as possible.[4] When measuring variable importance using the Gini index we are measuring how each variable used for splitting contributes to the homogeneity of the nodes and leaves in the random forest. Each time a node is split the Gini coefficient is calculated for the child nodes and this is compared to that of the parent node with the goal to create progressively more homogeneous nodes.[4] The Gini coefficient for a completely homogeneous node is 0 and for a completely heterogeneous node is 1, meaning that variables which contribute to high node homogeneity will have a high decrease in Gini coefficient. The changes in Gini coefficient elicited by each variable are summed and normalized and compared to each other determine relative variable importance.

Overall, when using out-of-bag error as the benchmark the goal is to produce a model with low error (high accuracy) and when using node purity as the benchmark the goal is to produce a model with pure or homogeneous nodes (all observations in the node having the same or similar predicted value). Variables can contribute to model accuracy, node purity, both, or neither metric. This contribution is measured relative to the other variables in the model meaning that the variable importance is ranked on a relative scale. It is important to note that there are no significance tests performed in these analyses.

**Table A.1.** Administrative data comorbidity case definitions

| **Variable** | **Case Definition** |
| --- | --- |
| Diabetes | Two OHIP claims within a 1-year period or one hospitalization related to diabetes or one ODB diabetes mellitus drug claim[5,6] |
| Hypertension | Two OHIP claims within a 2-year period or one hospitalization related to hypertension  OR one OHIP claim followed by a hospitalization related to hypertension within a 2-year period[7,8] |
| Atrial Fibrillation | One hospitalization or emergency department visit related to atrial fibrillation OR four OHIP claims within 1-year related to atrial fibrillation[9] |
| Myocardial Infarction | One hospitalization with most responsible diagnosis of acute myocardial infarction and no acute myocardial infarction hospitalization in the previous year[10]  Prior to the year 2016, this was collected in the Ontario Myocardial Infarction Dataset. Post 2016, the same definition was used to identify patients through the DAD. |
| Hospital Frailty Risk Score | Generated from the presence of 109 different ICD-10 codes in the two years prior to the index event. Full score available from Gilbert and colleagues (2018)[11] |
| Passive Surveillance Stroke seVerity Indicator (PaSSV) | Generated from the presence of 10 different severity markers during the first 48 hour of the patients index admission according to the methodology proposed by Yu and colleagues (2020)[12] |

OHIP: Ontario Health Insurance Plan; ODB: Ontario Drug Benefit Claims

**Table A.2.** Demographics of patients excluded due to privacy issues

| **Variable** | **Total (n = 202)** |
| --- | --- |
| Median Age (Q1, Q3) | 34 (24, 96) |
| Fiscal Year of admission (2 year groups), n (%)  2010 – 2011  2012 – 2013  2014 – 2015  2016 – 2017 | 37 (18.3%)  53 (26.2%)  52 (25.7%)  60 (29.7%) |
| Home Location, n (%)  Rural  Urban | 152 (75.2%)  50 (24.8%) |

**Table A.3.** Pairwise correlation of all covariates (Spearman’s Pairwise Correlation Coefficient)

|  | Frailty Score | PaSSV Score | Admission via Ambulance | Stroke Type | Age | Stroke Unit Care | Fiscal Year | Thrombolysis Use | Sex | Hypertension | Diabetes | Rural Home Location | Myocardial Infarction | Atrial Fibrillation | Income Quintile |
| --- | --- | --- | --- | --- | --- | --- | --- | --- | --- | --- | --- | --- | --- | --- | --- |
| Frailty Score | 1.00 | - | - | - | - | - | - | - | - | - | - | - | - | - | - |
| PaSSV Score | -0.21 | 1.00 | - | - | - | - | - | - | - | - | - | - | - | - | - |
| Admission via Ambulance | 0.19 | -0.45 | 1.00 | - | - | - | - | - | - | - | - | - | - | - | - |
| Stroke Type | 0.01 | 0.21 | -0.06 | 1.00 | - | - | - | - | - | - | - | - | - | - | - |
| Age | 0.23 | -0.06 | 0.18 | 0.06 | 1.00 | - | - | - | - | - | - | - | - | - | - |
| Stroke Unit Care | -0.01 | -0.03 | 0.07 | 0.07 | -0.02 | 1.00 | - | - | - | - | - | - | - | - | - |
| Fiscal Year | 0.04 | -0.03 | 0.01 | <-0.01 | <0.01 | 0.04 | 1.00 | - | - | - | - | - | - | - | - |
| Thrombolysis Use | <0.01 | -0.23 | 0.18 | 0.14 | -0.03 | 0.14 | -0.01 | 1.00 | - | - | - | - | - | - | - |
| Sex | -0.09 | 0.13 | -0.05 | -0.01 | -0.18 | 0.02 | 0.01 | <0.01 | 1.00 | - | - | - | - | - | - |
| Hypertension | 0.14 | -0.03 | 0.05 | 0.01 | 0.31 | -0.01 | 0.01 | -0.02 | -0.05 | 1.00 | - | - | - | - | - |
| Diabetes | 0.09 | 0.05 | -0.02 | 0.05 | 0.03 | -0.02 | 0.02 | -0.04 | 0.05 | 0.18 | 1.00 | - | - | - | - |
| Rural Home Location | -0.05 | 0.05 | -0.01 | 0.02 | <-0.01 | -0.07 | <-0.01 | <0.01 | 0.02 | -0.01 | -0.01 | 1.00 | - | - | - |
| Myocardial Infarction | 0.10 | <-0.01 | 0.03 | 0.04 | 0.06 | -0.01 | -0.01 | <0.01 | 0.06 | 0.09 | 0.10 | 0.01 | 1.00 | - | - |
| Atrial Fibrillation | 0.18 | -0.05 | 0.08 | 0.02 | 0.19 | -0.01 | -0.09 | <-0.01 | -0.04 | 0.11 | 0.02 | -0.01 | 0.08 | 1.00 | - |
| Income Quintile | -0.04 | <0.01 | -0.01 | -0.01 | 0.04 | -0.02 | -0.03 | 0.02 | 0.02 | -0.02 | -0.06 | <0.01 | -0.01 | 0.01 | 1.00 |

**Figure A.1.** Cohort selection. IKN = ICES key number

**
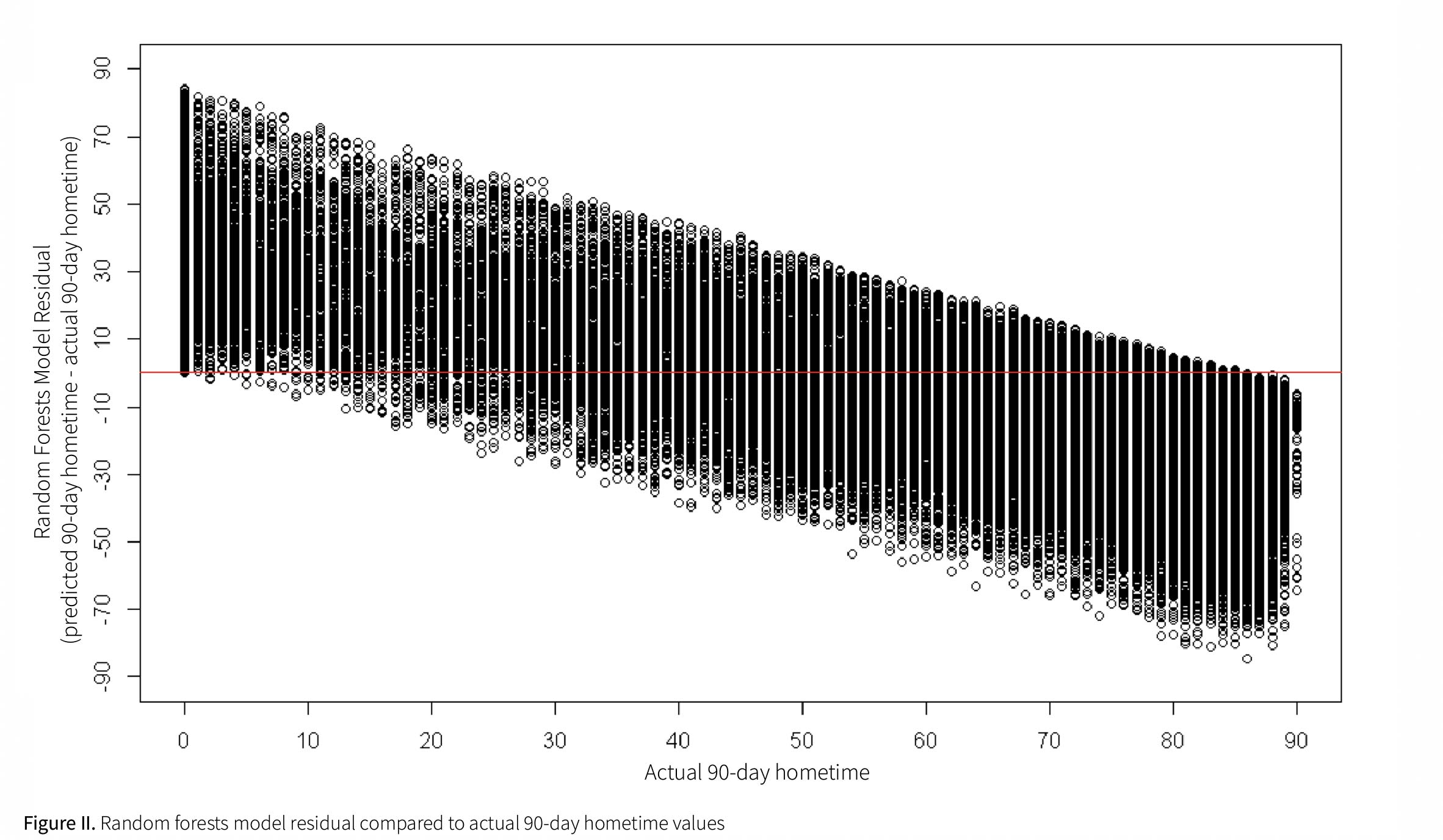
**

**Figure A.2.** Random forests model residuals compared to actual 90-day hometime values

**Supplemental References:**

1. Breiman L. Random Forests. Machine Learning. 2001;45:5–32.

2. Hastie T, Tibshirani R, Friedman J. The Elements of Statistical Learning. Springer Science & Business Media; 2013.

3. Cutler A, Cutler DR, Stevens JR. Random Forests. Ensemble Machine Learning. 2nd ed. Boston, MA: Springer US; 2012. pp. 157–75.

4. Breiman L, Friedman J, Stone CJ, Olshen RA. Classification and Regression Trees. Boca Raton: Routledge; 1984.

5. Lipscombe LL, Hwee J, Webster L, Shah BR, Booth GL, Tu K. Identifying diabetes cases from administrative data: a population-based validation study. BMC Health Serv Res. BioMed Central; 2018;18:316–8.

6. Hux JE, Ivis F, Flintoft V, Bica A. Diabetes in Ontario: determination of prevalence and incidence using a validated administrative data algorithm. Diabetes Care. American Diabetes Association; 2002;25:512–6.

7. Tu K, Campbell NR, Chen Z-L, Cauch-Dudek KJ, McAlister FA. Accuracy of administrative databases in identifying patients with hypertension. Open Med. 2007;1:e18–26.

8. Tu K, Chen Z, Lipscombe LL, Canadian Hypertension Education Program Outcomes Research Taskforce. Prevalence and incidence of hypertension from 1995 to 2005: a population-based study. Cmaj. CMAJ; 2008;178:1429–35.

9. Tu K, Nieuwlaat R, Cheng SY, Wing L, Ivers N, Atzema CL, et al. Identifying Patients With Atrial Fibrillation in Administrative Data. Can J Cardiol. 2016;32:1561–5.

10. Austin PC, Daly PA, Tu JV. A multicenter study of the coding accuracy of hospital discharge administrative data for patients admitted to cardiac care units in Ontario. Am. Heart J. 2002;144:290–6.

11. Gilbert T, Neuburger J, Kraindler J, Keeble E, Smith P, Ariti C, et al. Development and validation of a Hospital Frailty Risk Score focusing on older people in acute care settings using electronic hospital records: an observational study. Lancet. 2018;391:1775–82.

12. Yu AYX, Austin PC, Rashid M, Fang J, Porter J, Hill MD, et al. Deriving a Passive Surveillance Stroke Severity Indicator From Routinely Collected Administrative Data: The PaSSV Indicator. Circ Cardiovasc Qual Outcomes. 2020;13:e006269.
